# Evaluation of the national surveillance system for invasive meningococcal disease, Italy, 2015–2018

**DOI:** 10.1371/journal.pone.0244889

**Published:** 2021-01-08

**Authors:** Xanthi D. Andrianou, Flavia Riccardo, Maria Grazia Caporali, Cecilia Fazio, Arianna Neri, Paola Vacca, Luigina Ambrosio, Patrizio Pezzotti, Paola Stefanelli

**Affiliations:** Department of Infectious Diseases, Italian National Institute of Health (Istituto Superiore di Sanità), Rome, Italy; National Institute for Infectious Diseases Lazzaro Spallanzani-IRCCS, ITALY

## Abstract

Enhanced laboratory-based surveillance of invasive meningococcal disease (IMD) in Italy was only assessed indirectly by numerically comparing surveillance data cases with hospital discharge records (HDR). In this study, we evaluated the completeness, timeliness and sensitivity of the IMD surveillance in Italy from 2015 to 2018. Completeness and timeliness were described at the national and subnational level. A capture-recapture analysis was conducted to evaluate the sensitivity and positive predictive value (PPV) using HDR as the external source with a combination of deterministic and probabilistic approaches. The characteristics of the unmatched vs. matched cases were compared using multivariable Poisson modeling. Overall, the completeness of data improved, except for specific variables. Timeliness of notifications also improved to a median of 4 days from onset to reporting. For the years 2015–2017, the sensitivity of the surveillance was estimated at 71.4% and the PPV at 77.5%, changing to 80.6% and 66.9% respectively after removing cases with a secondary meningitis diagnosis. We noted substantial sub-national differences. In 2018 sensitivity was 66.5% (135/203) and the PPV was 79.4% (135/170). The adjusted relative risk of being unmatched in 2015–2017 was higher in cases that were ≥60 years, had missing information or symptom onset in December. The IMD surveillance system overall performs well with completeness and timeliness improving in time. Specific challenges identified for individual variables should guide further improvement. Notwithstanding limitations posed by the comparison database, sensitivity and PPV are promising. The study highlights that promoting etiological ascertainment in people ≥60 years and addressing sub-national challenges are the main current challenges to address.

## Introduction

The Gram-negative *Neisseria meningitidis* is transmitted from person to person and is the main causal agent for invasive meningococcal disease (IMD). IMD is a vaccine preventable disease and, according to the European Surveillance System data in 2017, 3221 confirmed IMD cases were reported from 30 EU/EEA member states, with highest rates reported in <1 year-olds, followed by 1–4 year-olds and adolescents and young adults (15–24 year-olds) [1, 2]. In Italy, the incidence of reported laboratory confirmed IMD cases was 0.3/100000 in 2017 and the vaccination is included in the National Immunization Plan which recommends vaccination with one dose of the serogroup C meningococcal vaccine for children aged 13–15 months-old, four doses of the serogroup B vaccine administered between 3 and 13 months and one dose of the tetravalent vaccine against serogroups A, C, W and Y for adolescents (12–14 years old) [3–5].

Italy is divided in 19 regions and 2 autonomous provinces that are responsible for planning and delivering health care services. This also includes the coordination and management of infectious diseases surveillance, with the support of the Ministry of Health and the Italian National Institute of Health (Istituto Superiore di Sanità, ISS). The surveillance of IMD in Italy is part of the National Surveillance System (NSS) for invasive bacterial diseases which also includes the surveillance of invasive cases from *Streptococcus pneumoniae*, *Haemophilus influenzae* and all other bacterial meningitis [6]. The system has been operating since 2007 with the objectives of: (i) monitoring the temporal and spatial distribution of cases, (ii) describing epidemic trends and circulating serogroups, (iii) estimating the number of cases that can be prevented, and (iv) identifying vaccination failures.

All suspected IMD cases are initially reported to the local health units and, after laboratory confirmation, they are reported a secure online platform which is coordinated by the ISS that is hosting the National Reference Lab (NRL) (S1 Fig in S1 File). Laboratory testing is conducted either at the regional or the national level at the NRL, if required.

Evaluation of the IMD NSS at the national level was conducted indirectly in Italy only in a study that compared the number of IMD cases reported to the NSS with hospital admission records over a period of 10 years [7]. This study showed an increasing agreement between the two sources of information during the study period and indicated that there is underreporting. A more detailed analysis of regional data was conducted by the Veneto region in another study that combined information from the NSS, the cases notified to the Ministry of Health, and the Laboratory Surveillance System of Veneto and compared them with the regional hospital discharge records (HDR) found a sensitivity of 76.7% [8]. The completeness of the different case characteristics included in the notification and the timeliness of the notifications, however, were not assessed nor described. At national level the completeness, timeliness and sensitivity of the IMD surveillance of IMD has not been previously evaluated directly. Therefore, the objective of this study was to evaluate these three aspects of IMD surveillance in Italy from 2015 to 2018.

## Methods

### Completeness and timeliness

All IMD cases reported at the NSS between 2015–2018 were used in the analysis. The surveillance attributes evaluated were the following:

- Completeness for variables that are important for public health action such as outcome and vaccination status, variables describing the basic case characteristics such as age, and sex, and variables describing clinical aspects such as comorbidities and sequelae. Additionally, we evaluated the completeness of the information on the serogroups. The percentage of missing data was described by year, overall (national level) and by region/autonomous province. In reporting of the cases, mandatory fields are considered complete even if they have been filled with the indication of “unavailable” or “unknown” information. Therefore, we also described the percentage of available information, defined as the percentage of cases for which the value is not “unavailable” or “unknown”.
- The timeliness for the different streams of information coming to the main platform. The time to first notification was calculated as the between symptom onset and first notification, that can be either to the local health unit or directly to the platform. Secondly, as some regions do not input the data directly to the platform but they send all relevant information (either in file form or the final datasets through certified and password protected mails) to be imported at the ISS, we calculated the delay of notification to the platform as the time between symptom onset and notification to the platform.

#### Capture-recapture analysis 2015–2018

To evaluate the sensitivity and the positive predictive value (PPV) of the IMD surveillance, we performed a capture-recapture analysis using hospital discharge records (HDR) as the external source. The HDR are routinely collected since 1995 and they include all hospitalizations in Italy, including dates of admission and discharge, basic demographics (e.g. sex, age) and diagnoses (primary and up to five secondary diagnoses) coded using the ninth edition of the International Classification of Diseases (ICD9). These data are collected independently from the surveillance data for other purposes. We assumed that, due to the severity of IMD, all cases reported to the surveillance system have been hospitalized. We compared the “surveillance cases” (cases that have been reported to the surveillance system) and “hospitalized cases” (cases that are included in the HDR with an ICD9 diagnosis coded as 036.XX). We used a combination of deterministic and probabilistic approaches. The capture-recapture analysis was conducted in two steps as data became available. The first step was an analysis of the surveillance data reported between 2015 and 2017 and the 2015–2017 HDR records. As soon the relevant data became available, in the second step, the analysis was repeated on surveillance data reported in 2018 and the 2018 HDR records.

*Capture-recapture step 1*: *2015–2017*. The capture-recapture analysis of the 2015–2017 IMD cases reported to the NSS was further divided in several parts. First, pairs of smaller datasets by region and sex, i.e. one dataset for the hospital cases and one for the surveillance cases, were created (n = 41). Then, the pairs were divided in three groups: (i) group 1: pairs of datasets of more than 10 cases, (ii) group 2: pairs of datasets where one of the two datasets had 10 or less cases, and (iii) group 3: pairs of datasets from the regions of Lombardy and Sicily for which hospital codes in the surveillance and hospital records were not based on the same index. The pairs of datasets of group 1, were processed using probabilistic record linkage (using the package fastLink in R [9, 10]). All matches with a post-probability of above 0.85 were retained and considered matched. The remaining, i.e. unmatched, records were re-categorized in pairs by region and sex. For the pairs of datasets of group 2 and group 3 matching was conducted manually. The remaining hospital cases were compared with cases of meningitis (ICD9 code 036.XX) retrieved from the 2005–2014 hospital records to exclude any case with a previous diagnosis that might have been coded as meningitis upon a new admission due to the medical history. In the next step, the unmatched surveillance records were compared with hospital records that had been coded as having an unspecified meningitis (ICD9 codes: 3229 or 3209). Sensitivity was estimated as the proportion of matched cases (found in both the surveillance and the hospital discharge records) to the total number of cases (i.e. hospitalized cases), while the PPV was estimated as the proportion of matched cases to the total number of assumed cases (i.e. surveillance cases). In a sensitivity analysis, we recalculated the sensitivity and the PPV excluding cases with a secondary diagnosis of meningitis from the unmatched hospital cases, and estimating the matched pairs excluding those for which the hospital case had a secondary diagnosis of meningitis.

*Characteristics of unmatched vs matched cases between the surveillance and the hospital records*, *2015–2017*. Characteristics of unmatched vs matched cases were compared by multivariable Poisson models with robust variance clustered by hospital code for both sources separately. In the model evaluating the unmatched vs matched surveillance IMD cases age, availability of exact information on the date of birth (no vs yes), sex, year of reporting, and month of onset symptoms were included; in the model evaluating the unmatched vs matched HDR cases, age, sex, year of discharge and month of admission were considered. In both models preliminary analysis evaluated up to 9 age groups (i.e., <1, 1, 2–4, 5–14, 15–24, 25–34, 35–44, 45–59, ≥60 years old); on the basis of log-likelihood ratio tests the final models included the following age-group: <15, 15–34, 35–59, ≥60 years old); regarding month of symptom onset (for IMD surveillance) or admission (for HDR cases) we initially explored the percentage of unmatched cases by month and then we grouped months as follows: July and August (summer holiday months), December and all the remaining months together.

*Capture-recapture step 2*: *2018*. For 2018, the cases of the surveillance and the HDR were compared manually. Initially, we screened all cases against the HDR records of cases with diagnosis of meningococcal disease and in the second step the unmatched surveillance records were compared with HDR entries that were coded as having an unspecified meningitis (ICD9 codes: 3229 or 3209). As in the analysis of step 1, the sensitivity and the PPV were estimated.

The analyses were performed in R (version 4.0.0) and in STATA (version 16).

### Ethical statement

This study was conducted using data from the Italian national surveillance of invasive meningococcal diseases that are collected in the hospitals for the routine and analysed within the mandate of the Italian National Institute of Health; therefore, no ethical approval was required [11]. The hospital discharge records include pseudoanonymized data provided for research purposes to the Italian National Institute of Health.

## Results

### Brief description of the invasive meningococcal disease cases reported in Italy, 2015–2018

A total of 784 IMD cases were reported in the period, from 189 in 2015 to 170 in 2018 (**Table 1**). The mean age of patients was 30.8 years in 2015, 29 years in 2016, 32.5 years in 2017 and 35.2 years in 2018, with the overall mean at 31.6 years. The male-to-female ratio of the cases was approximately 1:1 during the study period (**Table 1**). In 2015 and 2016 the majority of the cases were reported from the regions of Lombardy and Tuscany. Less populated regions such as Molise and the Aosta Valley reported zero or less than five cases during the study period.

**Table 1:** Characteristics and distribution by region/autonomous province of the invasive meningococcal disease cases reported to the national surveillance system and included in the evaluation, Italy, 2015–2017.

	2015	2016	2017	2018	Overall
N	189	227	198	170	784
Age (mean, sd)	30.8 (25.3)	29.0 (23.8)	32.5 (25.3)	35.2 (27.6)	31.6 (25.5)
Sex (%)					
Female	90 (47.6)	114 (50.2)	96 (48.5)	86 (50.6)	386 (49.2)
Male	98 (51.9)	113 (49.8)	102 (51.5)	84 (49.4)	397 (50.6)
Unknown	1 (0.5)	0 (0.0)	0 (0.0)	0 (0.0)	1 (0.6)
**Region/Autonomous province**					
Abruzzo	4 (2.1)	3 (1.3)	3 (1.5)	3 (1.8)	13 (1.7)
Basilicata	1 (0.5)	2 (0.9)	1 (0.5)	1 (0.6)	5 (0.6)
Calabria	1 (0.5)	3 (1.3)	0 (0.0)	0 (0.0)	4 (0.5)
Campania	11 (5.8)	32 (14.1)	21 (10.6)	21 (12.4)	85 (10.8)
Emilia-Romagna	14 (7.4)	18 (7.9)	24 (12.1)	8 (4.7)	64 (8.2)
Friuli-Venezia Giulia	2 (1.1)	2 (0.9)	1 (0.5)	2 (1.2)	7 (0.9)
Lazio	16 (8.5)	19 (8.4)	21 (10.6)	14 (8.2)	70 (8.9)
Liguria	2 (1.1)	5 (2.2)	9 (4.5)	18 (10.6)	34 (4.3)
Lombardy	34 (18.0)	45 (19.8)	33 (16.7)	35 (20.6)	147 (18.8)
Marche	2 (1.1)	8 (3.5)	3 (1.5)	3 (1.8)	16 (2.0)
Molise	0 (0.0)	0 (0.0)	1 (0.5)	0 (0.0)	1 (0.1)
Piedmont	9 (4.8)	16 (7.0)	14 (7.1)	5 (2.9)	44 (5.6)
Autonomous Province of Bolzano	5 (2.6)	1 (0.4)	5 (2.5)	2 (1.2)	13 (.7)
Autonomous Province of Trento	3 (1.6)	1 (0.4)	0 (0.0)	2 (1.2)	6 (0.8)
Apulia	12 (6.3)	5 (2.2)	8 (4.0)	5 (2.9)	30 (3.8)
Sardinia	4 (2.1)	5 (2.2)	4 (2.0)	9 (5.3)	22 (2.8)
Sicily	13 (6.9)	8 (3.5)	11 (5.6)	6 (3.5)	38 (4.8)
Tuscany	38 (20.1)	41 (18.1)	17 (8.6)	17 (10.0)	113 (14.4)
Umbria	4 (2.1)	0 (0.0)	4 (2.0)	6 (3.5)	14 (1.8)
Aosta Valley	2 (1.1)	1 (0.4)	0 (0.0)	0 (0.0)	3 (0.4)
Veneto	12 (6.3)	12 (5.3)	18 (9.1)	13 (7.6)	55 (7.0)

### Completeness and timeliness

Overall the completeness of the surveillance information as well as the content improved over the years. However, large variability was observed among the different variables. For example, the variables of age and sex had nearly no missing values, with the exception of a case in 2015 for which the sex was missing. On the other hand, the comorbidities and sequelae were two of the variables with the highest level of missing information. More specifically, for the comorbidities the percentage of available information was 24.3% in 2015. It increased to 37.4% in 2017 and decreased again to 34.1% in 2018. The percentage of available information on sequelae was below 10% in 2015 and increased (at 18.7%) in 2017. In 2018, the percentage of available information on sequelae reached 17.1%, remaining below 20% for all the years included in the study. Availability of information on serogroups also improved between 2015 and 2018. In 2018, serogroup data availability among all reported cases was 94.1% (**Table 2**).

**Table 2:** Summary of the completeness and timeliness of selected variables included in the surveillance of invasive meningococcal disease in Italy, 2015–2017.

	2015		2016		2017		2018	
	Completed fields (%)	Available information (%)	Completed fields (%)	Available information (%)	Completed fields (%)	Available information (%)	Completed fields (%)	Available information (%)
**Age**	100		100		100		100	
**Sex**	99.5		100		100		100	
**Outcome**	87.8	62.4	92.5	67.8	100	81.3	100	91.8
**Vaccination status**	100	65.6	100	67.0	100	73.7	100	64.1
**Comorbidities**	92.6	24.3	100	27.3	100	37.4	100	34.1
**Sequelae**	100	4.2	100	16.3	100	18.7	100	17.1
**Serogroups**		77.2		83.7		92.4		94.1
**Timeliness**	**Median number of days**		**Median number of days**		**Median number of days**		**Median number of days**	
**Date of symptom onset–Date of notification**	5		4		4		4	
**Date of symptom onset–Date of upload on the platform**	21		18		13		11	

The timeliness of notifications improved over time with a median number of days ranging from five, in 2015, to four, in 2018. The trend of improvement in timeliness of notifications was confirmed in most of the regions/autonomous provinces with overall less than seven days of median time from onset to notification (**Table 2**). Similarly, improvement was observed in the median time between symptom onset and upload of the patient information in the online platform (**Table 2**).

### Capture-recapture, 2015–2018

#### Capture-recapture step 1: 2015–2017

In total 613 IMD cases reported in the NSS were compared with 663 cases from the HDR in the first round of the analysis (S1 Table in S1 File) and additionally three hospital cases having a diagnosis of unspecified meningitis were added in later stages. One hospital case was excluded because it had been previously admitted (between 2005 and 2014) with a meningitis diagnosis. One case of the initial set of 614 surveillance detected cases was excluded from the analysis because the hospitalization had occurred in another country.

In total 472 surveillance and hospital cases were matched in the first and second round of the capture recapture from 41 pairs of datasets by region/autonomous province and sex. Overall, 260 cases were matched with probabilistic matching (first round) and 212 manually (second round). The remaining hospital records (n = 191) were compared with meningitis cases from the HDR (ICD9 code 036) diagnosed in 2005–2014 hospital records. One case from the remaining hospital records was removed in this step, leaving 190 unmatched hospital records. In the next step, three unmatched surveillance records were matched with hospital cases that had been coded as having an unspecified meningitis (ICD codes: 3229 or 3209). In total, 475 records were matched.

The sensitivity of the surveillance was estimated at 71.4% and the PPV at 77.5%. After removing 91 hospital cases those that had meningitis as a secondary diagnosis, and 65 matched pairs in which the hospital case had a secondary diagnosis of meningitis, the sensitivity was estimated at 80.6% and the PPV at 66.9% (Table 3).

**Table 3:** Summary of the capture-recapture analysis and the calculated indicators (i.e. sensitivity and positive predictive value).

	HDR	Surveillance	Matched	Unmatched HDR	Unmatched Surveillance	Sensitivity	Positive predictive value
	n	n	n	n	n		
Overall	665	613	475	190	138	71.4%	77.5%
*Secondary diagnosis*	156		65	91			
Sensitivity analysis*	509	613	410	99	203	80.6%	66.9%

*Assuming the hospital cases with secondary diagnosis were not included in the analysis.

The sensitivity varied by region/autonomous province. For example, in smaller regions in terms of population size and IMD incidence such as the Aosta Valley, Abruzzo, Molise and Basilicata all cases were matched (100% sensitivity). In other regions, e.g. Calabria, Umbria and Lazio the sensitivity was 40%, 45.5% and 51.8%, respectively (S2 Table in S1 File).

*Factors associated to unmatched cases both in IMD surveillance and in HDR*, *2015–2017*. Tables **4** and **5** show the estimated adjusted relative risk (ARR) of being unmatched in IMD surveillance and in HDR, respectively.

**Table 4:** Characteristics of reported Invasive Meningococcal Diseases (IMD) cases at the Italian enhanced surveillance system and Adjusted Risk Ratios (ARR) of being unmatched with IMD hospitalizations, Italy 2015–2017.

		Matched							
		yes	no		Total	ARR	95%CI		p-value
		n	n	%	n				
**Age**	***<15 (ref)***	153	31	16.8	184	1.00	-		
	***15–34***	147	47	24.2	194	1.41	0.98	2.03	0.07
	***35–59***	120	25	17.2	145	1.03	0.65	1.63	0.91
	***60+***	55	35	38.9	90	2.50	1.67	3.73	0.00
**Missing information on exact date of birth**	***yes***	451	121	21.2	572	1.00	-		
	***no (ref)***	24	17	41.5	41	1.95	1.21	3.13	0.01
**Sex**	***Female (ref)***	237	64	19.5	328	1.00	-		
	***Male***	238	74	22.0	337	1.18	0.86	1.61	0.31
**Year of reporting**	***2015 (ref)***	149	41	21.6	190	1.00	-		
	***2016***	168	58	25.7	226	1.27	0.87	1.86	0.21
	***2017***	158	39	19.8	197	1.01	0.68	1.51	0.95
Month of onset symptoms*	***January (ref)***	78	19	19.6	97	1.00	-		
	***February (ref)***	56	24	30.0	80				
	***March (ref)***	58	14	19.4	72				
	***April (ref)***	47	12	20.3	59				
	***May (ref)***	25	6	19.4	31				
	***June (ref)***	30	8	21.1	38				
	***July***	24	9	27.3	33	1.07	0.63	1.80	0.81
	***August***	28	4	12.5	32				
	***September (ref)***	28	6	17.6	34	1.00	-		
	***October (ref)***	31	7	18.4	38				
	***November (ref)***	43	9	17.3	52				
	***December***	27	20	42.6	47	1.95	1.21	3.13	0.01

Note: Some frequency numbers of matched cases by age group are in some cases slightly different from those reported in Table 5 because in the capture recapture analysis cases with different date of birth could be matched based on the other characteristics.

**Table 5:** Characteristics of hospitalizations for Invasive Meningococcal Diseases (IMD), characteristics and Adjusted Risk Ratios (ARR) of being unmatched with IMD Italian surveillance, hospital discharge registry, Italy, 2015–2017.

		Matched							
		yes	no		Total	ARR	95%CI		p-value
		n	n	%	n				
**Age**	***<15 (ref)***	152	30	16.5	182	1.00	-		
	***15–34***	148	37	20.0	185	1.23	0.77	1.97	0.39
	***35–59***	119	48	28.7	167	1.79	1.21	2.65	<0.01
	***60+***	56	75	57.3	131	3.63	2.44	5.40	<0.01
**Sex**	***Female (ref)***	237	91	27.7	328	1.00	-		
	***Male***	238	99	29.4	337	1.25	0.99	1.59	0.06
**Year of discharge**	***2015 (ref)***	149	53	26.2	202	1.00	-		
	***2016***	167	59	26.1	226	1.02	0.73	1.42	0.92
	***2017***	159	78	32.9	237	1.11	0.82	1.49	0.50
**Month of admission**	***January (ref)***	78	24	23.5	102	1.00	-		
	***February (ref)***	54	22	28.9	76				
	***March (ref)***	60	18	23.1	78				
	***April (ref)***	49	20	29.0	69				
	***May (ref)***	24	14	36.8	38				
	***June (ref)***	31	8	20.5	39				
	***July***	24	12	33.3	36	1.35	0.99	1.84	0.06
	***August***	29	15	34.1	44				
	***September (ref)***	24	9	27.3	33	1.00	-		
	***October (ref)***	34	15	30.6	49				
	***November (ref)***	41	15	26.8	56				
	***December***	27	18	40.0	45	1.42	1.01	2.00	0.04

Note: Some frequency numbers of matched cases by age group are in some cases slightly different from those reported in Table 4 because in the capture recapture analysis cases with different date of birth could be matched based on the other characteristics.

For the IMD surveillance cases, we found a significant higher ARR to be unmatched in cases aged ≥60 years old compared paediatric cases. Moreover, cases with missing information on the exact date of birth had a significant higher ARR of being unmatched compared to cases with complete information. With regards to the months of symptom onset, those who presented symptoms in December had significant higher ARR to be unmatched compared to those with symptom onset in the other months.

For the HDR cases, we found a significant and increasingly higher ARR of being unmatched for those aged 35–59 and ≥60 years old compared to paediatric cases and significantly higher ARR for those admitted in December compared to those admitted other months. It is of note that male cases and cases admitted in the summer period had higher ARR of being unmatched (compared to females and other months but December, respectively). However, this association was not strictly significant (p = 0.06).

#### Capture-recapture step 2: 2018

All 170 IMD cases reported in to the NSS in 2018 were compared with 202 cases from the HDR were included initially in the capture-recapture analysis for 2018. Overall, 134 cases were matched between the two data sources and another case of the surveillance was retrieved among HDR cases with unspecified meningitis. Therefore, for 2018, the sensitivity of the surveillance was 66.5% (135/203) and the PPV was 79.4% (135/170).

## Discussion

In this study we evaluated the completeness, timeliness, sensitivity and PPV of IMD surveillance in Italy, at national level. With regards to completeness, given that most of the fields are mandatory, therefore, the metric used in the evaluation was the availability of information. An improvement was noted especially in the variable of outcome, and on the vaccination status. With regards to timeliness, an improvement (i.e. decrease) was also noted in the median number of days between the onset of symptoms and the notification, as well as in the median number of days between the onset of symptoms and date of upload on the platform. Given the different approaches used by the different regions/autonomous provinces in sharing their surveillance forms on IMD, the longer lag between onset and date of upload in the platform, compared to the lab between onset and notification, was expected.

With regards to sensitivity and PPV, variation was observed between the regions/autonomous provinces. Sensitivity and PPV were estimated at 71.4% and 77.5% for at national level, assuming that the HDR include all IMD cases. Differences between regions/autonomous provinces were observed and they might be driving the national trend observed overall. Taking into consideration only the HDR cases with a primary diagnosis of IMD, the sensitivity of surveillance was estimated at 80.6%. However, the PPV decreased compared to the initially estimated 77.5% to 66.9%. These differences between the sensitivity and PPV of the main analysis and the sensitivity analysis indicate the limitations posed by using a comparison database of IMD cases, i.e. the HDR, that might not be the gold standard and might be the result of underreporting.

It should be noted that differences were observed in the estimated sensitivity and PPV by region/autonomous province for the study period. Regions, especially those with smaller population had sensitivity that reached 100% while in other cases the sensitivity was as low as 40%. One of the regions with the highest sensitivity (85.7%) was Tuscany, which experienced an IMD outbreak during the study period [12]. The regional differences might be the result of different workflows of reporting between the regions. The differences in sensitivity and specificity are also reflected in the timeliness differences that might also be due to the regional differences in the workflows of reporting until the cases reach the national level. The sensitivity for 2018 was lower compared to the pooled sensitivity for 2015–2017. The differences in sensitivity might indicate between year variability. The evaluation of the surveillance by pooling data from different years provides an overall assessment of the system, while the evaluation of specific years allows more targeted assessment and a snapshot of the quality of the system. In this manuscript we opted to use both approaches combining the past years and analysis the most recently available data, i.e. for 2018 separately. This approach allows also to enhance the communication between the national and the regional levels, to identify systematic or temporary differences between different regions and address specific issues that arise and might affect the timely notification of the cases. The evaluation of the surveillance of IMB in regular intervals e.g. yearly should be considered to allow for addressing gaps reporting of the cases.

Selecting the correct “gold standard” is the pitfall in capture-recapture studies. In Italy the surveillance is laboratory-based and therefore, the laboratory confirmed cases could not be used as a comparator against the surveillance cases, as it has been done in other countries. For example, in a study in Germany a comparison was made between the cases reported at the national level at the Robert Koch Institute (RKI) and the cases reported at the National Reference Center for Meningococci (NRCM) in 2013 [13]. The sensitivity for the RKI was 89% while the sensitivity for the NRCM was 65%. In Ireland, in a study published in 2018, the infectious diseases reporting system covered 87% of the laboratory reported cases [14]. In another study in South Africa, the comparison was again between national surveillance and the laboratory surveillance, and the authors report sensitivity under different scenarios that ranges between 98% and 99% [15].

The use of other sources of IMD cases besides the HDR to estimate the sensitivity of the IMD surveillance was not possible in this study. In a previous study from Veneto, one of the 19 Italian regions, the authors explored how complete three sources namely the mandatory MoH notifications, NSS (ISS notifications) and laboratory surveillance, were compared to the HDR records and the sensitivity of the three sources (combined) was estimated at 76.7% (the authors refer to it as completeness). In our analysis the sensitivity for Veneto was 61.5%. This is indicative of the added value regional analysis might have and the use of different sources in the comparison and in future capture-recapture analysis for the sensitivity of the surveillance. This alternative approach could allow us to propose specific and targeted interventions to improve the performance of the system.

In this analysis, we also evaluated factors associated to unmatching in the two data sources used i.e. the IMD surveillance and the HDR. For the IMD surveillance cases, the association between being an unmatched case and having a missing date of birth suggests that low data quality did not allow the matches to be found in the HDR. The higher ARR of being unmatched if symptom onset was in December may be due to reporting having taken place in the subsequent year. For example, those with symptom onset at the end of December 2017 could have been reported to the HDR of the 2018 and therefore they could not be matched due to the fact that the HDR records of 2018 were not at the time of the analysis available. People aged ≥60 years old had ARR of being unmatched in both archives, also the crude case numbers were similar in both data sources suggesting that the missed linkage for some of them could be related to low quality data about the variables used for the linkage.

The main limitation of the study is the use of a comparison database for the estimation of the sensitivity and PPV that might not be the gold standard. Given that the surveillance is based on laboratory confirmation and that not all surveillance cases could be matched with the HDR we can hypothesize that possible coding errors or admission of the cases in hospitals different that the ones that were included in the surveillance might have happened. Since the surveillance form did not include as a mandatory field the hospital of admission of the cases part of the unmatched surveillance cases could be attributed to errors in coding most likely from the part of the surveillance.

Given the results of this evaluation, several recommendations could be made to improve the surveillance of IMD in Italy. These recommendations include making some fields mandatory in the forms and maybe excluding some fields that are not very relevant for public health action. Additionally, emphasis should be placed in communicating and advocating for timely data importation by that showing the value of timely notification for public health action. Moreover, regular re-evaluation and assessment of the sensitivity of surveillance will be valuable to explore how the overall surveillance of IMD can be improved. Lastly, this evaluation can pave the way to engage more with the regions/autonomous provinces to identify more adequate “gold standard” databases for comparison and estimation of “local” sensitivity to propose tailored changes in their practices to improve surveillance.

## Supporting information

S1 File (DOCX)
