# Supplementary material for: Evaluation of the national surveillance system for invasive meningococcal disease, Italy, 2015–2018
Source: PLoS One. 2021 Jan 8;16(1):e0244889. doi: 10.1371/journal.pone.0244889 (PMC7793274; doi:10.1371/journal.pone.0244889)
Supplement: S1 File — (DOCX) [file pone.0244889.s001.docx]

## S1 File

**Serotyping and microbiological characterization**


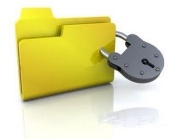


**Local Health Unit/Region**

**Fax, e-mail, phone (within 12h)**

**Disease notification**

**Data validation/integration (within 7 days); updates**

**Shipment of isolates/ clinical samples**

**Regional Reference Laboratory (if present) and National Reference Laboratory (ISS)**

**Password restricted access**

**Serotyping and microbiological characterization**


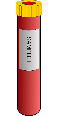

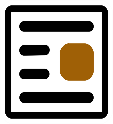


**ISS: periodic reporting (published online** [**www.iss.mabi.it**](http://www.iss.mabi.it/)**)**

**Data shared with the European Centre for Disease Prevention and Control (ECDC-Stockholm)**


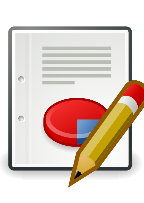

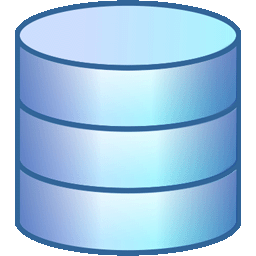


**Data analysis**

**Diagnosis***


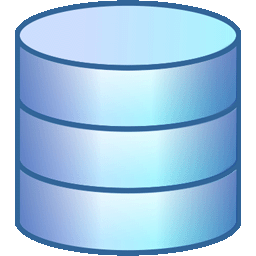


**Online IT platform- Surveillance of invasive bacterial diseases**

**coordinated by the ISS**

**Hospital**

**Laboratory**

**(confirmation)**

S1 Fig Structure and dataflow for the surveillance of invasive bacterial diseases in Italy.

**Hospital**

**Hospital**

**Ward**

**(clinical suspicion)**

**Serotyping and microbiological characterization**

*S1 Table Basic characteristics of the surveillance and hospital discharges records' cases compared in the capture-recapture analysis.*

|  | **Hospital discharges records cases (n=663)** | **Surveillance cases (n=613)** |
| --- | --- | --- |
|  | **n (%)** | **n (%)** |
| Year |  |  |
| 2015 | 201 (30.3) | 189 (30.8) |
| 2016 | 226 (34.1) | 227 (37.0) |
| 2017 | 236 (35.6) | 197 (32.1) |
| Sex |  |  |
| Female | 326 (49.2) | 301 (49.1) |
| Male | 337 (50.8) | 312 (50.9) |
| Age (mean (sd)) | 34.2 (26.1) | 30.7 (24.8) |

*S2 Table Number of cases by source, i.e. HDR and surveillance, and by region along with the sensitivity and PPV by region.*

| **HDR** |  |  |  |  |  |  |
| --- | --- | --- | --- | --- | --- | --- |
|  | **Total** | **Matched** | | **Unmatched** | | **Sensitivity** |
| **Region/Autonomous Province** | **n** | **n** | **%** | **n** | **%** |  |
| Piedmont | 38 | 31 | 81.6 | 7 | 18.4 | 81.58 |
| Aosta Valley | 2 | 2 | 100.0 | 0 | 0.0 | 100.00 |
| Lombardy | 110 | 80 | 72.7 | 30 | 27.3 | 72.73 |
| Autonomous province of Bolzano | 10 | 9 | 90.0 | 1 | 10.0 | 90.00 |
| Autonomous province of Trento | 5 | 3 | 60.0 | 2 | 40.0 | 60.00 |
| Veneto | 52 | 32 | 61.5 | 20 | 38.5 | 61.54 |
| Friuli-Venezia Giulia | 7 | 5 | 71.4 | 2 | 28.6 | 71.43 |
| Liguria | 22 | 15 | 68.2 | 7 | 31.8 | 68.18 |
| Emilia-Romagna | 55 | 46 | 83.6 | 9 | 16.4 | 83.64 |
| Tuscany | 91 | 78 | 85.7 | 13 | 14.3 | 85.71 |
| **Umbria** | **11** | **5** | **45.5** | **6** | **54.5** | 45.45 |
| Marche | 13 | 9 | 69.2 | 4 | 30.8 | 69.23 |
| **Lazio** | **85** | **44** | **51.8** | **41** | **48.2** | 51.76 |
| Abruzzo | 7 | 7 | 100.0 | 0 |  | 100.00 |
| Molise | 1 | 1 | 100.0 | 0 |  | 100.00 |
| Campania | 71 | 50 | 70.4 | 21 | 29.6 | 70.42 |
| Apulia | 27 | 22 | 81.5 | 5 | 18.5 | 81.48 |
| Basilicata | 4 | 4 | 100.0 | 0 | 0.0 | 100.00 |
| **Calabria** | **10** | **4** | **40.0** | **6** | **60.0** | 40.00 |
| Sicily | 34 | 21 | 61.8 | 13 | 38.2 | 61.76 |
| Sardinia | 10 | 7 | 70.0 | 3 | 30.0 | 70.00 |
|  |  |  |  |  |  |  |
| **Surveillance** |  |  |  |  |  |  |
|  | **Total** | **Matched** | | **Unmatched** | | **PPV** |
| **Region/Autonomous Province** | **n** | **n** | **%** | **n** | **%** |  |
| Piedmont | 39 | 31 | 79.5 | 8 | 20.5 | 79.49 |
| Aosta Valley | 3 | 2 | 66.7 | 1 | 33.3 | 66.67 |
| Lombardy | 111 | 80 | 72.1 | 31 | 27.9 | 72.07 |
| Autonomous province of Bolzano | 11 | 9 | 81.8 | 2 | 18.2 | 81.82 |
| Autonomous province of Trento | 4 | 3 | 75.0 | 1 | 25.0 | 75.00 |
| Veneto | 41 | 32 | 78.0 | 9 | 22.0 | 78.05 |
| Friuli-Venezia Giulia | 5 | 5 | 100.0 | 0 |  | 100.00 |
| Liguria | 16 | 15 | 93.8 | 1 | 6.2 | 93.75 |
| Emilia-Romagna | 55 | 46 | 83.6 | 9 | 16.4 | 83.64 |
| Tuscany | 97 | 78 | 80.4 | 19 | 19.6 | 80.41 |
| Umbria | 7 | 5 | 71.4 | 2 | 28.6 | 71.43 |
| Marche | 13 | 9 | 69.2 | 4 | 30.8 | 69.23 |
| Lazio | 56 | 44 | 78.6 | 12 | 21.4 | 78.57 |
| Abruzzo | 10 | 7 | 70.0 | 3 | 30.0 | 70.00 |
| Molise | 1 | 1 | 100.0 | 0 |  | 100.00 |
| Campania | 66 | 50 | 75.8 | 16 | 24.2 | 75.76 |
| Apulia | 25 | 22 | 88.0 | 3 | 12.0 | 88.00 |
| Basilicata | 4 | 4 | 100.0 | 0 |  | 100.00 |
| Calabria | 4 | 4 | 100.0 | 0 |  | 100.00 |
| Sicily | 32 | 21 | 65.6 | 11 | 34.4 | 65.63 |
| Sardinia | 13 | 7 | 53.8 | 6 | 46.2 | 53.85 |
